# Feasibility assessment of fixation patterns in multiple mandibular fractures: A clinical study

**DOI:** 10.6026/973206300221846

**Published:** 2026-03-31

**Authors:** Arshdeep Singh Kohli, Dharmendra Kumar M.G, Samir Mansuri, Sree Ram Subba Reddy Gudimetla, Gokul Venkateshwar, G. Kailasakumar Reddy, Rahul Tiwari

**Affiliations:** 1 Department of Oral and Maxillofacial Surgery, Luxmi Bai Institute of Dental Sciences and Hospital, Patiala, Punjab, India; 2 Department of Oral and Maxillofacial Surgery, C.K.S. Theja Dental College, Renigunta Road, Tirupati, Andhra Pradesh, India; 3 Department of Maxillofacial Surgeon, Al Kuwait Hospital, Dubai, United Arab Emirates; 4 Department of Oral Maxillofacial surgery, Sree Mithra Dental and MaxFace Specialists, Tanuku, Andhra Pradesh, India; 5 Department of Oral and Maxillofacial Surgery, D.Y. Patil University School of Dentistry, Nerul, Navi Mumbai, Maharashtra, India; 6 Department of Oral and Maxillofacial Surgery, Nimra Institute Of Dental Sciences, Ibrahimpatnam, Krishna, Andhra Pradesh, India; 7 Department of Oral and Maxillofacial Surgery, Narsinhbhai Patel Dental College and Hospital, Sankalchand Patel University, Visnagar, Gujarat, India

**Keywords:** Mandibular fractures, fixation sequence, condylar fracture, open reduction and internal fixation (ORIF), feasibility

## Abstract

Optimal fixation sequencing in multiple mandibular fractures involving the condyle remains uncertain and inappropriate. Sequence selection may increase the risk of postoperative malocclusion and occlusal instability. An analysis of a retrospective cohort of surgically treated case by occlusal stability (the nature in which the teeth make contact) for operative feasibility and postoperative complications, as well as fixation sequence and fracture pattern was conducted. Multifocal mandibular fractures were complex in clinical practice with condylar fracture detected in 40.6% of the patients. The body related 56.3% and symphysis/parasymphysis 51.0% were the most frequent sites. In the condyle first sequence, less postoperative occlusal discrepancies were observed.

## Background:

Comminuted fractures of the mandible is an intricate variation within the modality of maxillofacial Injuries by a reason of securing stable anatomical reduction for several functional units yet maintaining mandibular continuity and occlusion. Open reduction and internal fixation (ORIF) is most frequently performed, but the surgical complication is greatly increased when there is a corresponding concomitant condylar facture of the symphysis, parasymphysis, body or angle fracture [1, 2]. In such patterns, fixation sequence could influence precision of reduction, fixation stability and presence of a postoperative malocclusion or revision requirement [3]. Furthermore, complications including deep surgical site infection and implant failure remain in clinical focus especially for high-energy injuries and comminuted fractures [4]. Although since then modern plating systems and surgical strategies have improved results, no consensus has been found on the "ideal" pattern to be preferred in fixation of multifocal fractures of the mandible especially where condylar is included [5]. Therefore, it is of interest to report and evaluate the feasibility and early clinical outcomes of different fixation sequencing strategies in patients with multiple mandibular fractures, particularly those involving the condyle.

## Materials and Methods:

This was a retrospective clinical study which took place in a tertiary hospital based oral and maxillofacial surgery department. All data were extracted with ethical clearance granted by the institutional review committee. Patients with ≥18 years of age and presence of 2 or more mandibular fractures sites proven by computed tomography were included when treated by ORIF with titanium miniplates and/or load-bearing plates and observed for a minimum follow-up period of 6 months. Patients with pathologic fractures, pediatric patients and edentulous mandibles needing alternative fixation or panfacial trauma requiring complex reconstruction plates were excluded. Demographic data, mechanism of injury, site of fracture, condylar involvement, surgical approach used, type of fixation applied (length and number of screws), duration of surgery and postoperative outcome were noted.

Patients were divided into the following groups according to the sequence of fixation:

[1] Condyle-first series (reduction followed by fixation of the condyles) 

[2] Caudal-based (symphysis/body/angle fixation first).

The primary feasibility end points were operative time, difficulty in reduction during surgery and the need for occlusal adjustment during surgery. The clinical outcomes were malocclusion, infection, plate instability and the reoperation. Descriptive statistics and chi-square test, independent t-test were used in statistical analysis with level of significance at p<0.05.

## Results:

Table 1 (see PDF) Baseline demographics and fracture characteristics are outlined. The patients were predominately male (77.1%) and road traffic accidents were the most common cause of injury (53.1%). Multifocal mandibular fractures were complex in clinical practice with condylar fracture detected in 40.6% of the patients. The body 56.3% and symphysis/parasymphysis 51.0% were the most frequent sites. Three-site fractures that represent high injury severity were found in a third of the cases. Table 2 (see PDF) displays the difference in the results of the sequencings. Condyl-first group was less intraoperative occlusal adjusted (10.5% versus 24.1%) and was statistically significant (p=0.048). Condyl-first sequencing also significantly reduced postoperative malocclusion (5.3% and 13.8; p=0.041). The time taken during the operation was a bit less in condyl-first group, but not significant. Both groups had minimal infection and hardware problems, which are part of overall practicability of the two methods in any day-to-day clinical practice.

## Discussion:

This study examined the relevance of fixation order to multitrauma cases and discovered that in specific scenarios (i.e., condyle with either anterior or posterior side fracture pattern of the mandible); the condyle-first pattern of sequencing produced more favorable types of occlusive outcomes. Whereas both approaches were possible, the condyl-first approach was linked to considerably minimal changes in intraoperative occlusion and minimal postoperative occlusal complications. Occlusion is the most functionally appropriate outcome of trauma surgery in mandibular fractures. The issue of SSI is the result of ORIF of mandibular fractures, particularly in the high-energy trauma and tooth-bearing sections fractures. The incidence of infection in this group of patients was moderate and comparable in groups, which is in line with evidence to confirm that postoperative infection in repair of mandibular fractures when up-to-date guidelines are adopted is rare [6, 7]. Similarly, the loosening and preoperative rates of hardware were low, hence proving the mechanical stability of the modern osteosynthesis systems. The sequencing issue is more important in cases with combination of condylar fractures and symphysis or angle fractures. In these instances, fixation of the tooth-bearing segments prior to restoration may "lock" malposition if condylar relationship is not re-established, which could result in a greater chance for occlusal discrepancy. This is in agreement with more recent contemporary analyses which have emphasized that surgical approach in condylar-associated multiple fractures should focus on reconstitution of condyle height and mandibular position to achieve goal occlusal ends [8, 9]. There is also a large body of evidence highlighting the fact that ORIF for condylar fractures can result in good functional outcomes, if it is carried out properly; however, choice of approach and surgeon experience would be critical [10, 11]. Biomechanically, in the mandible tension and compression are on opposite sides of the fracture fixation. Current validated biomechanical models provide further evidence for the fact that stability of fixation depends on fracture side and direction of load and therefore a personalized approach or strategy rather than one size fits all [12]. Furthermore, modern clinical series comparing plating methods in the management of mandibular angle fractures also show plate orientation to play a significant role in stability and complication rates [13, 14], such that the present paper indirectly supports the argument of sequenced intervention and consideration of plate in multi-site fractures. It can be said that condylar-first sequence is especially prospective when dealing with multiple fractures of condylar nature and this is because condylar instability was minimized. This happens to be in line with the growing current consensus that in the event that the condylar height and hence mandibular alignment are at stake, one should not delay condylar reduction. But on severely displaced or comminuted anterior fractures the possible rationale of a caudal-first repair is to provide stability of the mandibular base prior to addressing condylar issues. Limitations to this study include the retrospective nature, possible surgeon-dependent selection bias in sequencing and lack of long-term functional measures including mandibular range of motion or validated patient-reported outcome scores. Prospective comparative studies in the future May help. The development of evidence-based sequences would also benefit from having 'functional' endpoints standardised [15].

## Conclusion:

Fixation sequencing in patients with multiple mandibular fractures is feasible using either a condyle-first or caudal-first approach. However, in fracture patterns involving the condyle, a condyle-first strategy was associated with significantly fewer intraoperative occlusal adjustments and a lower incidence of postoperative malocclusion. The two methods showed low complication rates and satisfactory initial results. On the whole, an individualized pattern-based sequencing approach can benefit the stability of a fracture in the occlusal and a better functional rehabilitation in the early stages after ORIF of multifocal and mandibular fractures.
